# Ln-MOF in production of durable antimicrobial and UV-Protective fluorescent cotton fabric for potential application in military textiles

**DOI:** 10.1038/s41598-024-84020-z

**Published:** 2025-01-07

**Authors:** Hossam E. Emam, Reda M. Abdelhameed, Osama M. Darwesh, Hanan B. Ahmed

**Affiliations:** 1 Department of Pretreatment and Finishing of Cellulosic Fibers, Textile Research and Technology Institute, National Research Centre, 33 EL Buhouth St., Dokki, Giza, 12622 Egypt; 2 Applied Organic Chemistry Department, Chemical Industries Research Institute, National Research Centre, 33 EL Buhouth St., Dokki, Giza, 12622 Egypt; 3 Agricultural Microbiology Department, National Research Centre, 33 El-Buhouth St., Dokki, Cairo, 12622 Egypt; 4 Chemistry Department, Faculty of Science, Helwan University, Ain-Helwan, Cairo, 11795 Egypt

**Keywords:** Cotton, Ln-MOF, Cationization, Fluorescent, UV protection, Antimicrobial, Durable, Surface assembly, Organic-inorganic nanostructures, Synthesis and processing

## Abstract

Industrialization of military textiles faces many challenges and some requirements such as durability, protection and suitability for hostile environment must be provided. Herein, fluorescent protective cotton with ultraviolet radiation (UVR)-protection and antimicrobial property was currently prepared via the immobilization of lanthanide-metal organic framework (Ln-MOF). Cotton fabrics were primarily activated via cationization process with 3-Chloro-2-hydroxypropyltrimethyl ammonium chloride to obtain the cationized cotton (Q-cotton). Subsequently, Ln-MOFs based on Europium (Eu) and Terbium (Tb) were separately immobilized within cotton and Q-cotton fabrics. The obtained Ln-MOF@fabrics showed good fluorescent character, while three and four emission bands were estimated for Eu-MOF@fabric and Tb-MOF@fabric, respectively, related to the electron transition from 5D_0_ to 7F_0-4_ in Eu^3+^ and from 5D_4_ to 7F_3-6_ in Tb^3+^. After Ln-MOF incorporation, UVR-protection factor (UPF) was significantly enlarged from 1.9 (insufficient UPF) to 22.1–25.6 (good UPF) without cationization and to 32.4–37.8 (very good UPF) for Q-cotton. Against three different pathogens (*Escherichia coli*, *staphylococcus Aureus* and *Candida albicans*), Ln-MOF@fabrics exhibited good microbial reduction of 68–79% and 81–91% in case of cotton and Q-cotton, respectively. The cationization improved the functionality and durability of fabrics, while the acquired functions were still existed even after 10 repetitive washings.

## Introduction

Different textile materials are wide-scaled applicable in the military purpose, like the industrialization of “uniforms”, “protective clothes”, “socks”, “gloves”, “sheets”, “sweaters”, “sand bags”, among the others. In addition to the conventional requirements such as fastness, many progressions with textile industry are still needed to be extensively considered, in order to protect, to promote less stressing, injury and improve the soldier performances. Therefore, in spite of the fact that modern warfare is considerably increased to face the remote meanings, combat arms still essential. The protection developments as a function, like maintaining the correct heating environments, is importantly required for the body survival, the body protection against abrasive injury, windings, light, electric effects, toxicity and microbiological attack, are also considerably interested^1^. Protective and military textile materials were the major growing segments of smart textile materials. Protective textile materials could be ascribed as the textiles that could be applied for protecting of the human body from any external attacking. Serious safety factors were correlated to the applicability as well as the disposal of the chemical reagents that acted for their contemporary treatments. Therefore, the researchers were considered with the field of textile functionalization that were continued to look for alternative techniques as cost effective, environmentally safe and to produce textiles characterized with high fastness, without showing any effects on the degree of comfort for the produced clothes with enhancing the efficiency and the as-required resistance^2–6^.

Numerous approaches were considered with the investigation of new technologies for the acquirement of different add-functions for the textile materials, such as, “coloration”^7^, “self-cleaning”^8^, “optical characters”^9^, “insect repellency”^10^, “microbicide performance”^11^, “electromagnetic interference (EMI) resistance”^12–14^, and “ultraviolet protection action”^11,15–17^. Moreover, air-protective masks were prepared for protecting from the toxic gases via the immobilization of different chemicals, such as the lipophilic /highly efficient active carbons^18^. Some approaches were also considered with the application of some organic reagents for preparation of protective textile materials, such as triclosan for antibacterial action, benzo-phenone for ultraviolet resistance, di-methylol di-hydroxy ethylene urea for wrinkle resistance, fluorocarbons for lipophilicity, long-chain hydrocarbon and poly-dimethyl-siloxane for flexibility and high comfort^19,20^. Butane tetra-carboxylic acid, citric acid and maleic acid^21–23^ were also successfully exploited for acquiring of the textile materials wrinkle resistance.

Impregnation of metal organic frameworks (MOFs) as one of the highly adsorptive reagents within the textile materials for production of protective textiles is described as a challenge for study and investigation. Inclusion of metal organic framework, as one of the highly adsorbent materials within the textile matrix for preparation of protective textiles, is ascribed as a challengeable field of study. MOFs were exploited as modifiers to enhance the performance of textile materials. Since 2017, few researching reports were studied the application of MOFs for improving the UV-protection action of textiles^11,24,25^, water absorbency, antimicrobial action, and permeability. Also, MOFs were shown to be exhibited with no toxic effects on the human body^11^.

The point of novelty in the current approach is to investigate the affinity of Ln-MOF for multi-functionalization of cotton fabrics via immobilization of metal organic frameworks-based on Tb and Eu. Ln-MOF were successfully immobilized within cotton and cationized cotton (Q-cotton) in one-pot technique. The prepared fabrics were well-analyzed characterized via scanning electron microscope, energy dispersive X-ray, X-ray diffraction and infrared spectroscopy. The photoluminescent properties, UV-protection and antimicrobial were all evaluated for the produced textiles. The mechanical properties and the durability were both examined.

## Experimental section

### Materials and chemicals

Europium nitrate (Eu(NO_3_)_3_.5H_2_O, 99.9%), terbium nitrate (Tb(NO_3_)_3_.5H_2_O, 99.9%), 1,2,4-benzentricarboxylic anhydride (BTC, C_9_H_4_O_5_, 97%), methanol (CH_3_OH, ≥ 99.8%, anhydrous), 3-Chloro-2-hydroxypropyltrimethyl ammonium chloride (C_6_H_12_NOCl_2_, 60 wt%), acetic acid (CH_3_COOH, glacial, ≥ 99.7%), sodium hydroxide (NaOH, 99%), were all supplied from Sigma-Aldrich and used without further purification.

Bleached plain-woven 100% cotton fabric (150 g/m^2^, with 34 and 31 threads per cm along warp and weft directions, respectively) were kindly supported by El-Mahalla Company for Spinning and Weaving, El-Mahalla El-Kubra – Egypt and used as received without further treatments.

### Preparation processes

#### Cationization of cotton fabrics

Cotton fabrics were formerly activated by cationization process with the quaternary ammonium salt, using pad-dry-cure method according to the previous reports^26–28^. Cotton fabrics were alkalized by impregnation in 5% sodium hydroxide solution (wt/vol) for 15 min and then squeezed for removing the excess solution of alkali. The alkalized cotton was immersed in 30% 3-Chloro-2-hydroxypropyltrimethyl ammonium chloride (wt/vol) for 2 min and then gently squeezed using padder to get 100% wet pick-up. The fabrics were dried in oven at 75 °C for 10 min followed by curing process at 120 °C for 4 min. The unreacted cationizing agent was removed by washing twice with tap water and then the treated fabrics were neutralized by 1% acetic acid (wt/vol). Finally, the obtained cotton fabrics (namely, Q-Cotton) were dried at 75 °C prior to functionalization.

#### Synthesis of Ln-MOF@fabrics

Ln-MOF (Eu-MOF and Tb-MOF) were directly synthesized within fabrics (cotton and Q-cotton) by solvothermal process. In a beaker, 0.45 g of metal salt (Eu(NO_3_)_3_ or Tb(NO_3_)_3_) was individually added to 1.9 g of 1,2,4-nenzenetricarboxylic anhydride and 0.08 g NaOH and then the mixture was dissolved in 100 mL of deionized methanol/water (1:1). After complete dissolution, the mixture was then transported to the reactor of stainless-steel reactor lined with Teflon and then the specimens of fabrics were completely submerged in the mixture. The reactor was tightly closed and placed into an oven at 140 °C. After 48 h, the fabrics were taken out, washed twice with deionized water to remove the excess of Ln-MOF white deposits and then dried at 70 °C. The obtained fabrics were observed with white color and labeled as Eu-MOF@cotton and Tb-MOF@cotton for cotton and Eu-MOF@Q-cotton and Tb-MOF@Q-cotton for Q-cotton.

Characterization

The surface properties were investigated for all fabrics before and after modification with Ln-MOFs using high resolution scanning electron microscope (HRSEM Quanta FEG 250 FEI Company – Netherlands). While, the elemental analysis and elemental mapping were measured by using the energy dispersive X-ray analyzer (EDAX AME-TEK) unit which attached with the applied microscope. The infrared spectra for all-modified cotton fabrics (Eu-MOF@Cotton, Eu-MOF@Q-Cotton, Tb-MOF@Cotton and Tb-MOF@Q-Cotton) were recorded by using the infrared JASCO (FT/IR-4700 spectrophotometer-Japan). While, the attenuated total reflection (ATR)–Fourier transform infrared spectra (FTIR) unit was used to detect the transmission spectra in the common range of 500–4000 cm^−1^ with 2.0 cm^−1^ interval and 64 repetitive scans.

The diffraction of X-ray for the all obtained cotton fabrics (Eu-MOF@Cotton, Eu-MOF@Q-Cotton, Tb-MOF@Cotton and Tb-MOF@Q-Cotton) were determined by using Philips X’Pert MPD diffractometer. While, the measurements were carried out at room temperature (Cu, λ = 1.5406 Å) in the diffraction angles (2θ°) range of 10–70°.

The thermal stabilities of the all-cotton fabrics (Eu-MOF@Cotton, Eu-MOF@Q-Cotton, Tb-MOF@Cotton and Tb-MOF@Q-Cotton) was examined by the thermogravimetric analysis (TGA) using the STD Q600 V20.9 Build 20. While, the decomposition of TGA was evaluated up to 400 ℃ through using heating rate of 10 ℃/min.

The mechanical properties (ultimate strength and elongation at break) for the cotton fabrics (Eu-MOF@Cotton, Eu-MOF@Q-Cotton, Tb-MOF@Cotton and Tb-MOF@Q-Cotton) were measured by using the Asano machine MFG Co – Japan. While, the measurements were performed attributing to the reference standard method of ASTM method D2256 − 66T. Two measurements were performed for each fabric sample and the mean values were only concerned with the standard deviation.

The photographical images of the cotton fabrics (Eu-MOF@Cotton, Eu-MOF@Q-Cotton, Tb-MOF@Cotton and Tb-MOF@Q-Cotton) were picked up inside the isolated box containing UV lamp (input 220 V AC and output 4 W). The photos were taken by cell phone camera of Oppo A31 at short wavelength of 265 nm. The photoluminescent properties for the cotton fabrics (Eu-MOF@Cotton, Eu-MOF@Q-Cotton, Tb-MOF@Cotton and Tb-MOF@Q-Cotton) were detected through measurement the fluorescence emission. While, the emission spectra were determined at the excitation wavelength of 270 nm by Jasco FP-6500 spectrofluorometer – Japan (150 W Xenon lamp, 1800 grooves/mm emission monochromator).

The ultraviolet radiation (UVR) protection over the obtained fabrics (Eu-MOF@Cotton, Eu-MOF@Q-Cotton, Tb-MOF@Cotton and Tb-MOF@Q-Cotton) was estimated through measurement the UVR transmission (T %) through the fabrics. While, the transmission was performed by using the JASCO V-750 spectrophotometer – Japan, in the wavelength range of 200–400 nm with 2 nm intervals. The different factors presented in the blocking in UV-A region (UVA, 315–400 nm), the blocking in UV–B region (UVB, 280–315 nm) and the UV protection factor index (UPF), were all estimated by using the standard AATCC test 183–2010.44 method. For each tested sample, the measurements were carried out twice at different measurement areas and the average was only considered.

The antimicrobial action for the all-fabrics (Eu-MOF@Cotton, Eu-MOF@Q-Cotton, Tb-MOF@Cotton and Tb-MOF@Q-Cotton) was carried out against three different pathogenic microorganisms which formerly obtained from the American type culture collection (ATCC; Rockville, MD, USA), through using the standard quantitative method of shaking flask test^29–31^. The tested microorganisms were identified as *Escherichia coli* ATCC-25,922 (*E. Coli*), *staphylococcus Aureus* ATCC- 47,077 (*St. Aureus*) and *Candida albicans* ATCC-10,231 (*C. Albicans*) as –ve gram bacterial pathogen, as + ve gram bacterial pathogen and fungal pathogen, respectively. Firstly, the tested microbial pathogens were individually dispersed in the medium on the nutrient agar and maintained at 4 °C. 70 µL from each microbial suspension were added to 10 mL of the nutrient broth medium and then discs were transferred to inoculated tubes. Afterwards, the tubes containing bacterial strains and fungal strains were incubated at 37 °C and 28 °C for 24 h with shaking, respectively. At the end of required time, the optical density of samples was measured by JASCO UV 630 spectrophotometer at 550 nm and the reduction in the viable microbe was estimated through using the intensity of absorbance.

## Results and discussion

### Modification of cotton fabrics

The cellulosic fabrics (cotton) were firstly activated by cationization process with the quaternary ammonium salt according to literature. Fabrics were activated by soaking in sodium hydroxide (5%, wt/vol) for 10 min followed by squeezing to remove the excess solution. The alkalized fabrics were then padded in 3-Chloro-2-hydroxypropyltrimethyl ammonium chloride (50%, wt/vol) two times and squeezed to obtain a 100% wet pick-up. The treated fabrics were dried for 10 min at 80 °C followed by curing at 140 °C for 4 min. The cationized fabrics were washed with tap water and acetic acid (1%) for neutralization, then washed two times with tap water followed by drying at 80 °C.

**Fig. 1:**
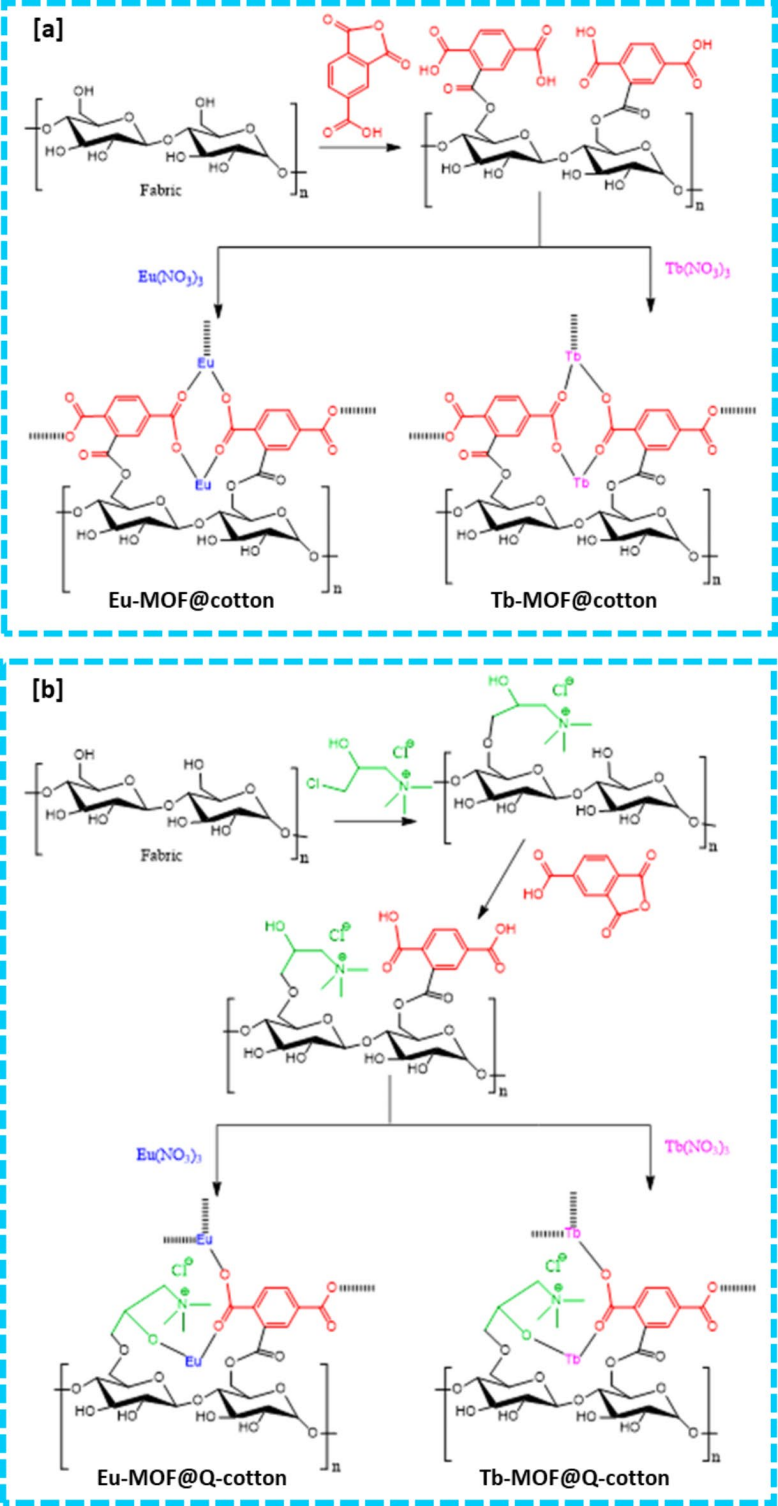
Schematic preparation of (**a**) Ln-MOF@Cotton and (**b**) Ln-MOF@Q-Cotton.

Cellulosic fabrics were firstly functionalized with quaternary amine to produce cationized fabrics as presented in Fig. 1. In the presence of sodium hydroxide, cellulosic fabrics were activated through the dissociation of the alcoholic groups. By addition of quaternary amine, the primary alcoholic group at C6 of cellulose was reacted with 3-Chloro-2-hydroxypropyltrimethyl ammonium via substitution reaction in the presence of alkali, giving cationized viscose (Fig. 1). Afterwards, Ln-MOFs were directly incorporated within fabrics by one-pot process. By addition of metal salt and organic linkers, substitution reaction is suggested to be proceeded between hydroxyl in cellulose and metal ions in MOF and Ln acted as a crosslinker between organic ligand and cellulose/cationized cellulose. Moreover, coordination and hydrogen bonding can be formed between metal (Ln) and functional groups (OH, NH) of MOF and hydroxyl groups (OH)/N of cellulose/cationized cellulose^9,32^. For the cationized fabrics, the amount of incorporated Ln-MOF was higher due to the cationization process and the quaternary amine salt acted as crosslinker between fabrics and Ln-MOF. The interaction between cationized fabrics and Ln-MOF become more available through cationization and increasing the functional groups of cellulose.

### SEM

**Fig. 2:**
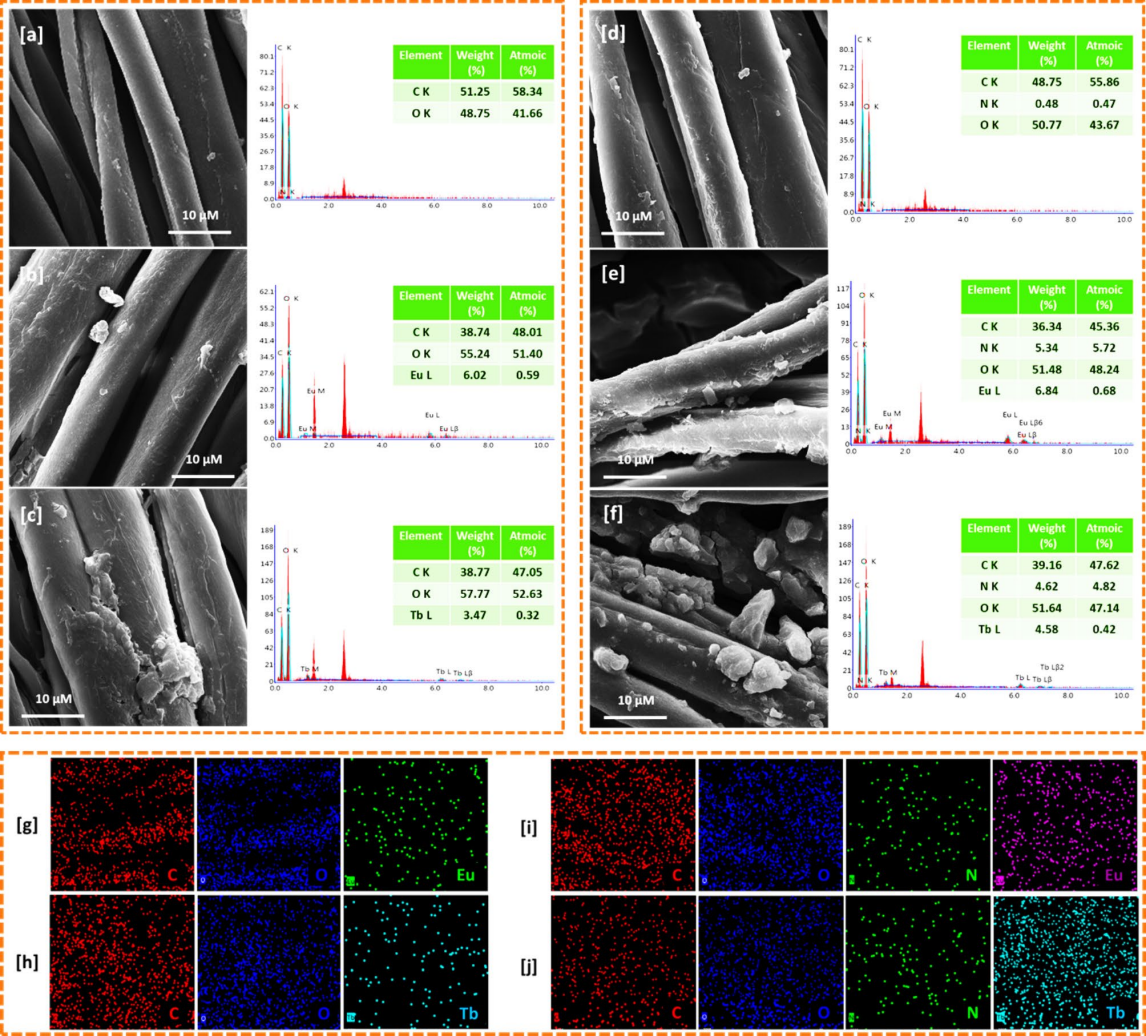
(**a–f**) Scanning micrographs and elemental analysis of EDX for Ln-MOF@Cotton; (**a**) Cotton, (**b**) Eu-MOF@Cotton, (**c**) Tb-MOF@Cotton, (**d**) Q-Cotton, (**e**) Eu-MOF@Q-Cotton and (**f**) Tb-MOF@Q-Cotton. (**g-j**) Mapping of elemental analysis of EDX for Ln-MOF@Cotton; (**g**) Eu-MOF@Cotton, (**h**) Tb-MOF@Cotton, (**i**) Eu-MOF@Q-Cotton and (**j**) Tb-MOF@Q-Cotton.

The structure and stability of the fabric composite is quite important through investigation the interfacial properties between the material of Ln-MOF and cotton^33,34^. Figure 2 represented the micrographs, EDX and elemental mapping for the cationized and Ln-MOF@fabrics. After the treatment with the cationizing agent, particles of the quaternary amine were observed over the clean/smoothed surface of the cotton fabrics. While, the nitrogen signal for the quaternary amine was clearly seen beside the signals of carbon and oxygen for the cotton. The results showed that; micro-sized Ln-MOF (Eu-MOF and Tb-MOF) were densely deposited over the surface of cotton fabrics forming Ln-MOF@fabrics. The geometrical shapes for the deposited Ln-MOF were changed with changing the metal from Eu to Tb. The deposited amounts of Ln-MOF onto the cotton fabrics were significantly increased after cationization process. The elemental analysis confirmed the deposition of Ln-MOF onto the fabrics, as the signals of Eu and Tb were individually recorded beside the signals of O, C and N for the cotton/Q-cotton fabrics. Additionally, the elemental analysis data confirmed that the deposited amounts of Ln-MOF was much higher in case of cationized cotton. This is reasonable due to the surface activation of the fabric after cationization through the increment in active sites which able to interact with Ln-MOF. As seen from the elemental mapping, well distribution of Eu & Tb elements onto the cotton fabrics were clearly observed, which reflected the good distribution of Ln-MOF over the cotton. Furtherly, the distributed Ln elements was significantly higher over the cationized cotton, which explained the role of cationization in increasing the content of Ln-MOF within fabrics.

### FTIR

**Fig. 3:**
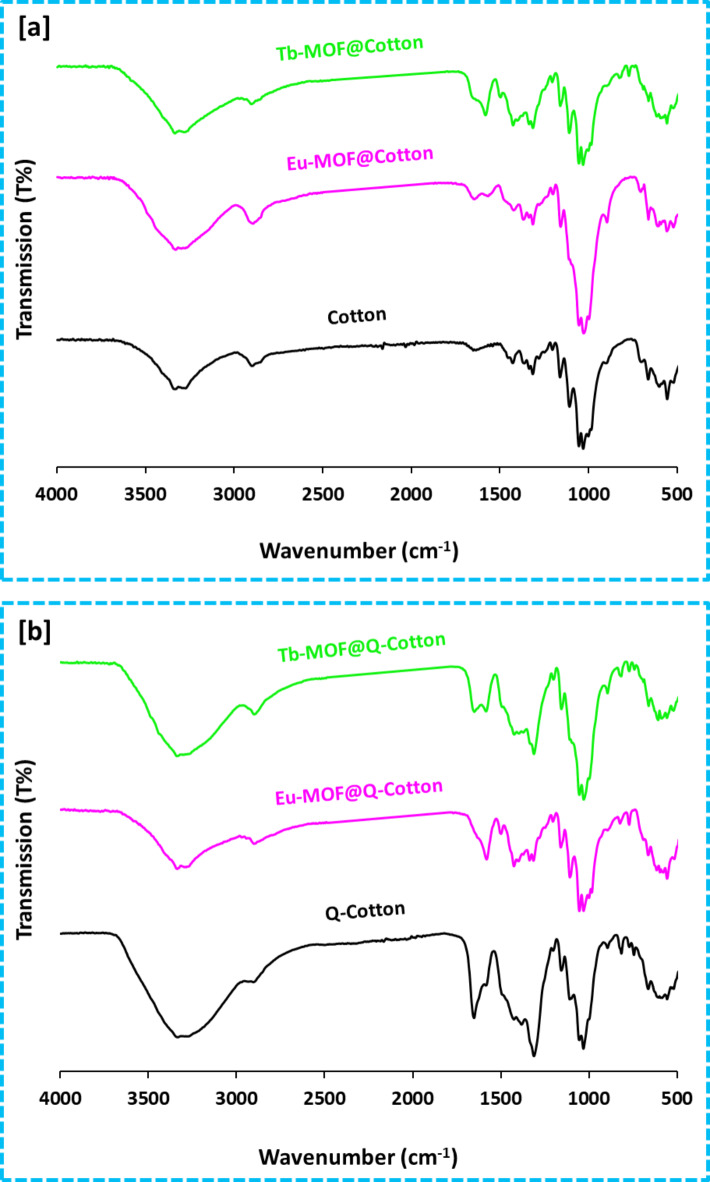
Infrared spectra for Ln-MOF@Cotton; (**a**) Cotton and (**b**) Q-Cotton.

The chemical interaction between the cotton/Q-cotton fabrics and the incorporated Ln-MOFs was investigated via changing in the functional groups (position and intensity of spectral peaks) which can be detected by the infrared spectra in Fig. 3. Cotton as cellulosic fabrics, showed several significant transmission peaks at 3292/3328, 2887/2890, 1643/1632, 1024/1022 and 893/896 cm^− 1^. The detected peaks are corresponded to the hydroxyl group (OH stretching), the aliphatic methyl (CH_2_ asymmetric stretching), the carbonyl (C = O stretching), the bending of C-O and the β-glycosidic linkage C-C, respectively^9^. After cationization, two peaks were clearly observed at 1643 and 1313 cm^− 1^, referring to the C-N stretching and N-H stretching, respectively^35^. These beaks declared the interaction between cotton and quaternary ammonium salt and consequently confirmed the formation of cationized cotton. By modification with Ln-MOF, three new transmission peaks were obviously recorded at 1580–1584 and 818–824 and 763–773 cm^− 1^ which are referred to the carboxylate group (COO stretching) of the organic acid ligand (BTC), the Ln-N and Ln-O, respectively^36,37^. The intensity of COO- group observed with higher intense in case of cationized cotton related to the higher content of Ln-MOF and this is agreed with the microscopic observation and elemental mapping.

### XRD

**Fig. 4:**
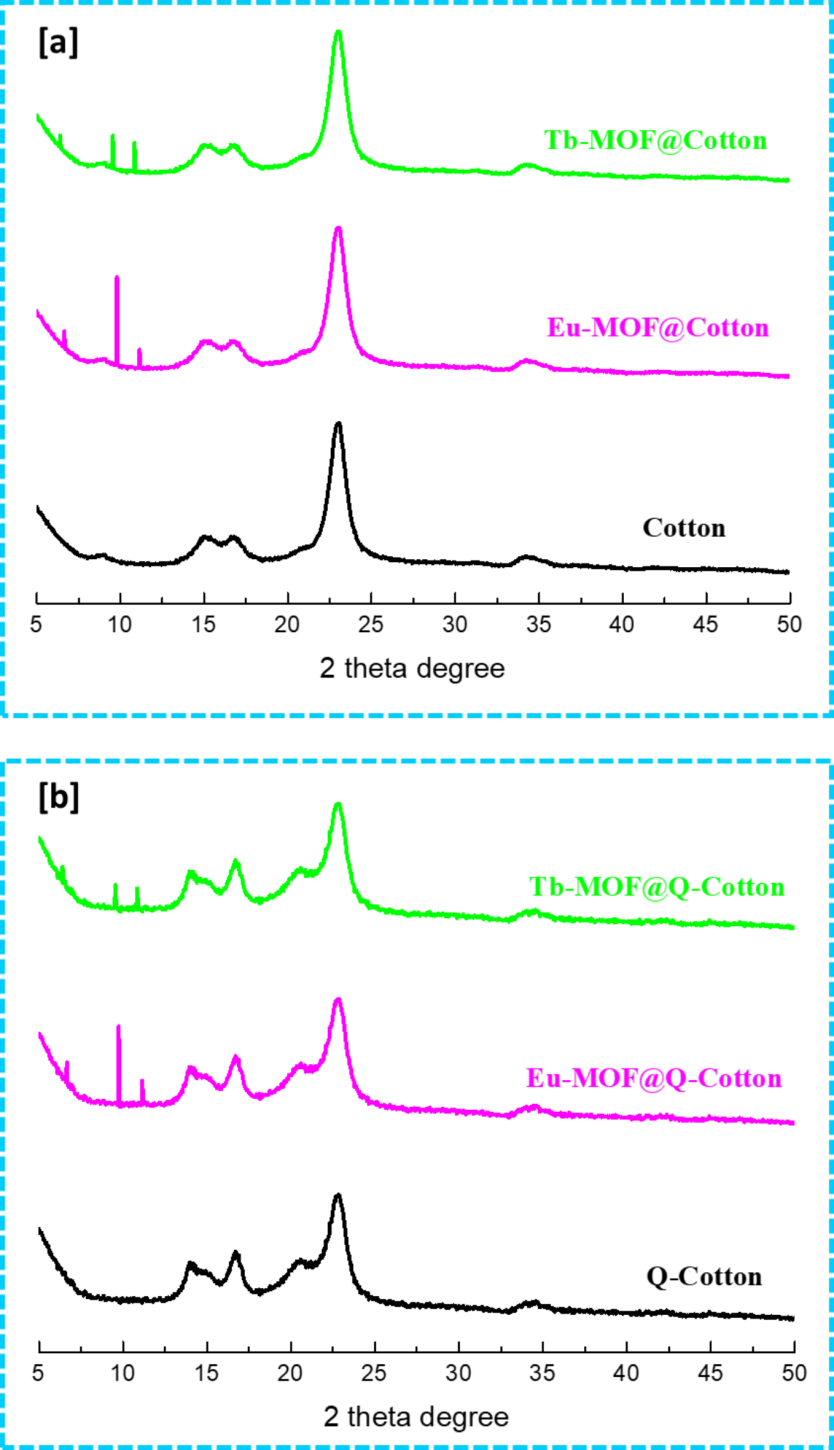
X-ray diffraction for Ln-MOF@Cotton; (**a**) Cotton and (**b**) Q-Cotton.

To further check the crystalline structure of cotton/cationized cotton before and after the modification with Ln-MOF, the XRD diffractions were optimized as shown in Fig. 4. Cotton and cationized cotton showed three significant diffractions at 2θ = 12.4°, 16.6° and 22.4°, which are corresponded to the crystalline structure of cellulose I^38^. After modification with Ln-MOF, the intensity of recorded diffractions for cellulose were reduced which reflected in the decrement in the crystalline structure of cellulose by Ln-NOF treatment, but with marginal effect which not affected on the wearable properties. Additionally, new diffractions were clearly recorded at 2θ = 9.7° & 11.3° and 2θ = 9.5° & 10.8° for Eu-MOF@fabric and Tb-MOF@fabric, respectively. The new obtained diffractions are characterized for the typical crystalline matrix of Eu-MOF and Tb-MOF, respectively, which are in harmony with the former studies^9,39^. Compared to cotton, the diffraction peaks of Ln-MOF are obviously seen with more intense in case of cationized cotton which might be related to higher Ln-MOF content as suggested by the observations of micrographs. Consequently, the diffraction results supported the observations of microscopic data for the successful incorporation of Ln-MOF within the cotton fabrics, without any serious deterioration in cellulose crystallinity.

### Thermal properties

**Fig. 5:**
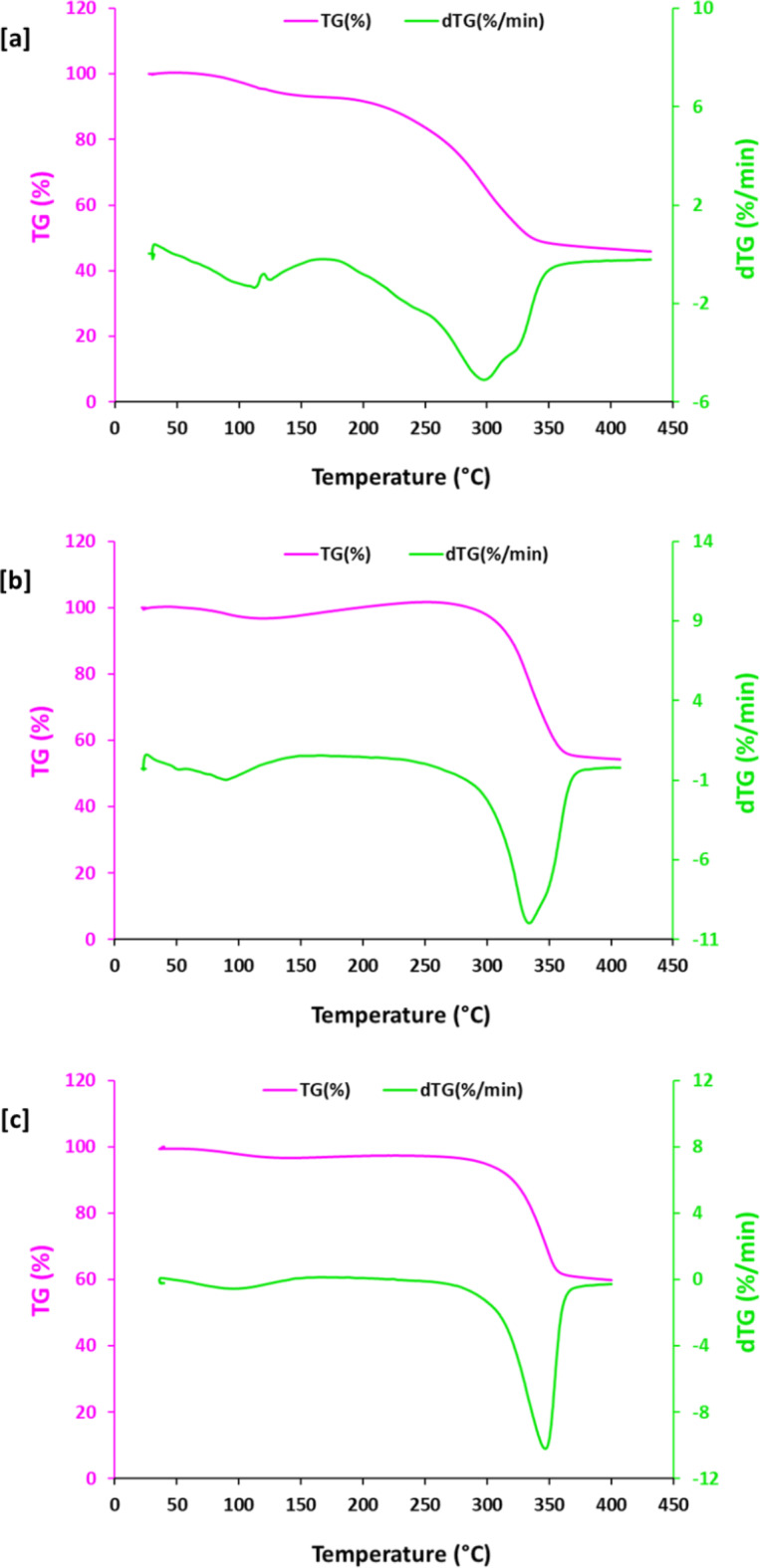
Thermal gravimetric analysis for Ln-MOF@Cotton; (**a**) Q-Cotton, (**b**) Eu-MOF@Q-Cotton and (**c**) Tb-MOF@Q-Cotton.

The effect of Ln-MOF incorporation on the thermal properties for cationized cotton fabrics was performed through investigation the thermal gravimetric analysis (TG, %) and profile of derivatization (dTG, %/min). The results in Fig. 5 notified that there were two stages in the weight loss of all tested fabrics (Q-cotton and Ln-MOF@Q-cotton). The weight loss in cationized cotton was detected after 190 °C, while, Ln-MOF@Q-cotton showed the thermal loss in weight at higher temperature of 300–310 °C. Consequently, the stable state of the weight loss of Q-cotton and Ln-MOF@Q-cotton was recorded. Subsequently, the insertion of Ln-MOF is resulted in significant lowering of weight loss in Q-cotton which reflected in the role of Ln-MOF in the enhancement of thermal stability for Q-cotton. At elevated temperature upto 310 °C, the weight loss for fabrics was considerably reduced from 40.5% for Q-cotton to 5.4% and 9.4%, in case of Eu-MOF@Q-cotton and Tb-MOF@Q-cotton, respectively. The estimated derivatization of TG declared that, the rate of loss in weight at 300 °C, was decreased from 5.3%/min for Q-cotton to 2.1%/min for Eu-MOF@Q-cotton and 2.4%/min for Tb-MOF@Q-cotton. The obtained data revealed that the incorporation of Ln-MOF was accompanied with the stability in thermal properties of cotton fabrics. This may be explained due to the successive domination of Ln-MOF through its converting to lanthanide oxides, and subsequently stabilized the fabrics at high temperature conditions^40,41^. Cotton incorporated with Eu-MOF exhibited rationally higher thermal stability compared to that treated with Tb-MOF.

### Mechanical properties

The effect of Ln-MOF immobilization on the mechanical properties (tensile strength; kg/cm^2^ and elongation at break; %) of the cotton fabrics were tested as summarized in Table 1. After cationization, the tensile strength of cotton was slightly enhanced, while the elongation at break was slightly decreased. The cationizing agent may act as a crosslinker between cellulosic chains and consequently improve the tensile strength. After Ln-MOF incorporation, the mechanical properties for cotton fabrics were reduced, while tensile strength was diminished from 167.2 kg/cm^2^ to 141.6–143.5 kg/cm^2^ for cotton and from 177.3 kg/cm^2^ to 158.8–161.5 kg/cm^2^ for Q-cotton. The data revealed that the tensile strength of fabric was marginally reduced by 14.2–15.3% and 9.0–14.4% for cotton and Q-cotton after Ln-MOF incorporation. While the elongation at break was lowered by 16.7–20.3% for cotton and 6.0–7.3 for Q-cotton due to the immobilization of Ln-MOF. The data reflected that the mechanical properties of fabrics was affected by Ln-MOF incorporation rather than that for the cationized fabrics. The marginal reduction in mechanical properties of fabrics could be attributed to the effect of Ln-MOF in breaking some of the inter-hydrogen bonds in the polymeric chains of cellulose^42^. However, compared to blank cotton, the mechanical properties of Ln-MOF@Q-fabrics are quite good and sufficient to the wearable cloth.

**Table 1:** The mechanical properties for Ln-MOF@Cotton fabrics.

Fabric	Ultimate strength (Kg/cm^2^)	Elongation at break (%)
**Cotton**	167.2 ± 5.2	13.8 ± 0.5
**Eu-MOF@Cotton**	143.5 ± 4.6	11.5 ± 0.6
**Tb-MOF@Cotton**	141.6 ± 5.5	11.0 ± 0.5
**Q-Cotton**	177.3 ± 8.2	12.4 ± 0.7
**Eu-MOF@Q-Cotton**	158.8 ± 7.1	11.8 ± 0.4
**Tb-MOF@Q-Cotton**	161.5 ± 5.8	11.5 ± 0.5

### Ultraviolet radiation protection

**Fig. 6:**
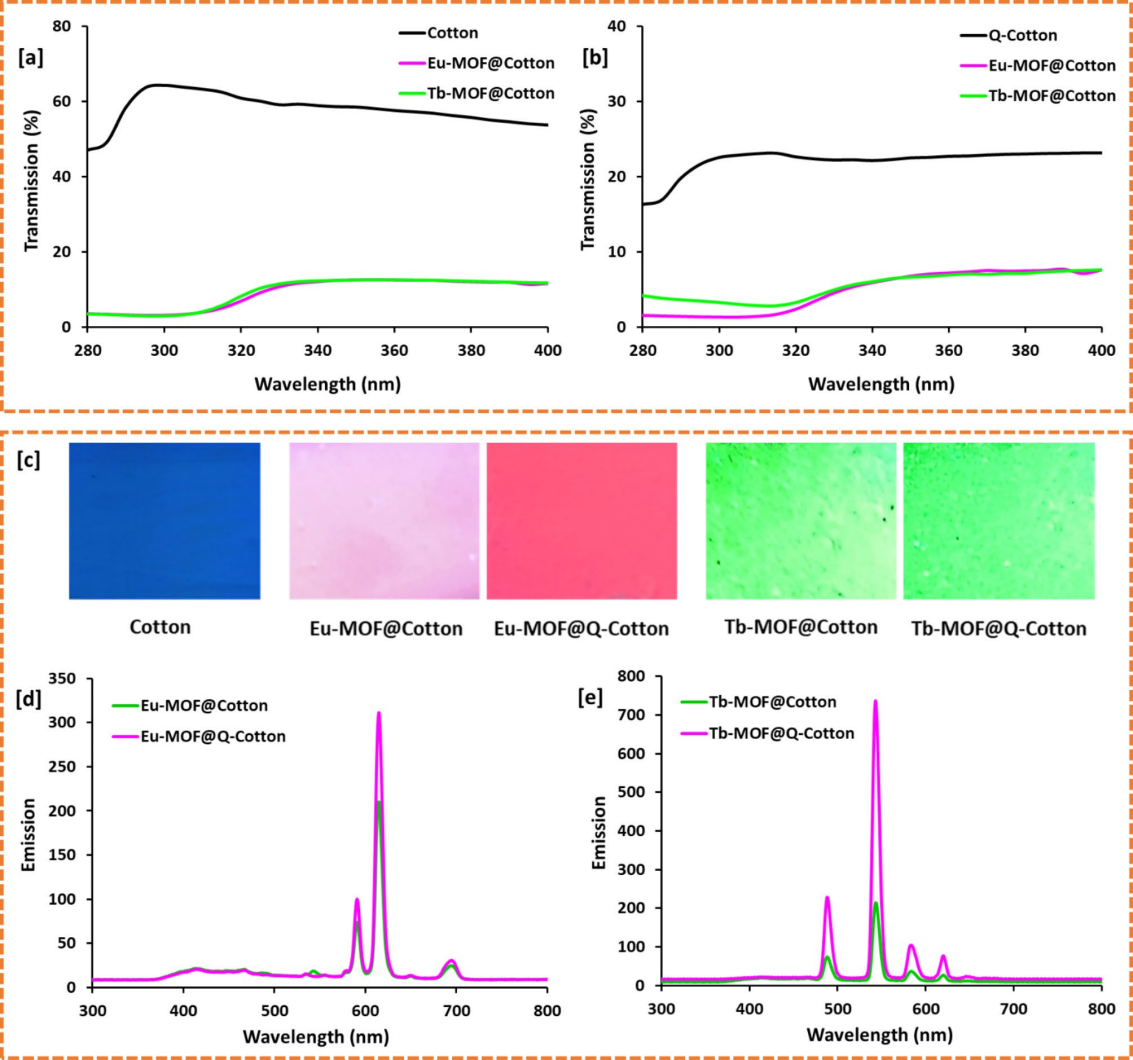
(**a**, **b**) UV transmission radiation through Ln-MOF@Cotton; (**a**) Cotton and (**b**) Q-Cotton. (**c**) Photographic images of Ln-MOF@cotton under UV lamp (short wavelength 254 nm). (**d**, **e**) Emission spectral results for the Ln-MOF@Cotton (at λ_ex_ = 270 nm) at room temperature; (**c**) Eu-MOF and (**d**) Tb-MOF.

Cotton is one of the most applicable textile materials because of its characteristic properties presented in breathability, comfortability and biocompatibility. However, cotton fabric has disadvantage of insufficient protection from UV radiation (UVR)^43,44^. Hence, the protection of cotton fabrics from UVR is highly required and several works were recently focused on the applications of different materials in UV-protective textiles^11,28,32,43^. Therefore, the protection properties of cotton fabrics before and after incorporation of Ln-MOF, was tested against UVR. The percentage of UVR-transmission (T%) was firstly recorded for the fabrics using the standard method of the Australian/New Zealand (AS/NZ) and the results are shown in Fig. 6(a, b). The ultraviolet protection factor index (UPF), T% in UV-A (315–400 nm) and T% in UV-B (280–315 nm) were all evaluated from data of T% and collected in Table 2.

**Table 2:** The UV protection properties (AATCC) for Ln-MOF@Cotton fabrics.

Sample	UPF	UV-A (T%)	UV-B (T%)	UPF rating
**Cotton**	1.9	59.2	57.1	*Insufficient*
**Eu-MOF@Cotton**	25.6	3.9	11.9	*Good*
**Tb-MOF@Cotton**	22.1	4.2	12.1	*Good*
**Q-Cotton**	5.5	20.9	22.6	*Insufficient*
**Eu-MOF@Q-Cotton**	37.8	1.5	6.7	*Very good*
**Tb-MOF@Q-Cotton**	32.4	3.1	6.6	*Very good*

The obtained data in Table 2 declared that, T% was dramatically lowered from 47.0 to 53.7% for cotton and 16.3–23.1% for Q-cotton to 3.5–11.8% Ln-MOF@cotton and to 1.6–7.6% for Ln-MOF@Q-cotton. After incorporation of Ln-MOF, the estimated UV-A (UV-B) were significantly decreased from 59.2% (57.1%) to 3.9–4.2% (11.9–12.1%) and from 20.9 (22.6%) to 1.5–3.1% (6.7–6.6%) in case of cotton and Q-cotton, respectively. The evaluated protection factor (UPF) was obviously enlarged from 1.9 to 22.1–25.6 for cotton and from 5.5 to 32.4–37.8 for Q-cotton after immobilization of Ln-MOF. According to the AS/NZ rating, the insufficient protection of cotton fabric was turned to good and very good UV protection after Ln-MOF incorporation in case of cotton and Q-cotton, respectively. There was no observable difference in UV-protection for fabrics after incorporation of Eu-MOF or Tb-MOF. Compared to Ln-MOF@cotton, Ln-MOF@Q-cotton was exhibited with higher UV-protection which may be related to higher content of Ln-MOF. The protection properties of Ln-MOF@fabrics against UVR is attributed to the incorporated Ln-MOF which is acted in reflection of UVR^11,24^.

In comparable overview with the previous studies, Ln-MOF@Q-cotton exhibited better UV protection than that obtained for textiles modified with metal nanoparticles^31,45,46^, metal salts^47^, metal oxides^48,49^, and carbon nanostructures^26,28^. While, similar UV protection properties were detected for TiO_2_/SiO_2_@fabrics^50^ and Pd@dyed cotton^27,31,45^. In case of textiles modified with different MOFs, viscose and cotton exhibited very good – excellent UV protection due to immobilization of Ce-MOF, Ti/En-MOF (MIL) and Ni-MOF (ZIF)^11,32,51^, which are quite close to that obtained here for cotton modified with Ln-MOF.

### Photoluminescent properties

Photoluminescent (PL) materials are widely applied in many things including toys, paper currencies, passport, sporting goods, watches, tickets, cards, bank cheques/notes and etc^52^. PL textiles are recently required for special purposes such as military textiles (policemen’s and soldier’s uniform) for differentiating between policemen and criminal or enemy^9,28,53^. Several materials were employed in textile applications including nanostructures^28,54,55^, hetero-compounds^53^ and conducting polymers^56^ and dyes/pigments^57–59^. Therefore, the photoluminescent properties for the Ln-MOF@cotton were investigated through measuring the emission spectra for the fabrics at the excitation wavelength of 270 nm and the results were presented in Fig. 6(d, e). Moreover, the photographical images for cotton before and after incorporation of Ln-MOF were shown in the UV chamber (Fig. 6c). When fabrics excited at short wavelength 254 nm, neither emitted color was observed for the pristine cotton nor cationized cotton under the UV-lamb. While Eu-MOF@fabric and Tb-MOF@fabrics were visually seen with red and green emitted color, respectively. Darker emitted color was observed for Ln-MOF@Q-cotton fabrics.

The spectral data showed that quite small broad band at 440 nm attributing to the host of cotton fabrics (since it is clearly seen in the un-modified samples). For Eu-MOF@cotton, three significant emitted bands were clearly detected at 592 nm, 614 nm and 698 nm. While, four characteristic bands were appeared at 490, 545, 586 and 622 nm in case of Tb-MOF@cotton. In case of Eu-MOF@fabric, the emission spectra are signified to the electron transitions of 5D_0_ to 7F_0 − 4_ in the Eu^3+^ ions. The three recorded emission bands (at 592 nm, 614 nm and 698 nm) are attributed to the three-transition series of from 5D_0_ to 7F_1_, from 5D_0_ to 7F_2_ and from 5D_0_ to 7F_4_, respectively^9^. For the Tb-MOF@fabrics, the four observed emission peaks are characterized for the electron transition of 5D_4_ to 7F_3 − 6_ in the Tb^3+^ emission. While, the recorded emission peaks (at 490, 545, 586 and 622 nm) are corresponded to the transition of electrons in the Tb from 5D_4_ to 7F_6_, from 5D_4_ to 7F_5_, from 5D_4_ to 7F_4_ and from 5D_4_ to 7F_3_, respectively^9,60^. The recorded emission intensity was higher in case of cationized fabrics which can be due to the higher loading amount of Ln, while the emission of Tb-MOF was rationally higher compared to Eu-MOF.

Designing of photoluminescent textiles was formerly performed by incorporation different materials such as metal nanoparticles^55^, metal salts (Zn^+ 2^, ZnS, SrAl_2_O_4_)^61–64^, lanthanides (In, Eu, Dy and Tb) containing composites^9,64–66^, hetero-compounds^53^ and carbon nanostructures^26,28^. Comparable to the all-aforementioned studies, the fluorescent fabrics obtained in the current work showed quite higher fluorescence intensity and more durable, consequently, they could be interestingly applied in the military textiles such as policeman uniform or solider clothes.

### Antimicrobial properties

The biological activities for the obtained Ln-MOF@fabrics were evaluated via investigation the microbial reduction against three different pathogens of *E. Coli* (G-ve bacteria), *St. Aureus* (G + ve bacteria) and *C. Albican* (Fungi). The data in Table 3 showed that the untreated cotton and Q-cotton fabric didn’t exhibit any microbial reduction regardless to the tested microbes. Ln-MOF@cotton fabrics showed good reduction in bacterial and fungal pathogens with percentage of 68–79% and 73–77%, respectively, which explained by the role of incorporated Ln-MOF. Much higher reduction (81–91%) in all tested microbial pathogens were observed in case of Ln-MOF@Q-cotton fabrics which may be related to the higher Ln-MOF content compared to Ln-MOF@cotton. The highest microbial reduction was recorded against *St. Aureus* rather than *E. Coli* and *C. Albican*, while there no significant difference in the microbial reduction between Eu-MOF@fabrics and Tb-MOF@fabrics. The results concluded that the immobilization of Ln-MOF is resulted in acquiring antimicrobial property for cotton fabric towards the bacterial and fungal pathogens, while cationization process furtherly improved the microbial reduction of cotton fabrics.

**Table 3:** Antimicrobial activity (reduction, %) for Ln-MOF@Cotton fabrics.

Sample	G-ve Bacteria	G + ve Bacteria	Fungi
	E. Coli	St. Aureus	C. Albican
**Cotton**	0	0	0
**Eu-MOF@Cotton**	68	75	73
**Tb-MOF@Cotton**	71	79	77
**Q-Cotton**	0	0	0
**Eu-MOF@Q-Cotton**	81	91	86
**Tb-MOF@Q-Cotton**	84	89	88

Based on the former studies focused on the antimicrobial properties for MOF or MOF containing composites^67–71^, the activity of Ln-MOF against the microbial pathogens, may be corresponded to the metal center of Ln (Eu or Tb) as well as the active functional groups of carboxylates. While, metal ions (Ln^+ n^) have an affinity in corruption of the ionic balance and consequently disruption of the cell membrane followed by metal ion penetration in the cytoplasm of microbe and finally leading to the deterioration of nucleic acid and cell proteins. Additionally, liberation of the reactive oxygen species (ROS) from terminal functional groups in MOF is also affected in the deterioration of cell wall in microbial pathogen. As analogous with antimicrobial textiles applied in literature such as metal (Ag, Au, Pd) nanoparticles^14,31,45,46,72^ or metal oxides (Cu_2_O, TiO_2_, ZnO)^48,49,73–78^, much better antimicrobial performance was exhibited by the currently prepared Ln-MOF@cotton fabrics. Compared to MOF (ZIF, Cu-BTC, Ce-MOF)@textiles^11,51,79^, quite similar microbial protection data was evaluated for Ln-MOF@fabrics, however different measurement methods were applied.

### Durability

**Fig. 7:**
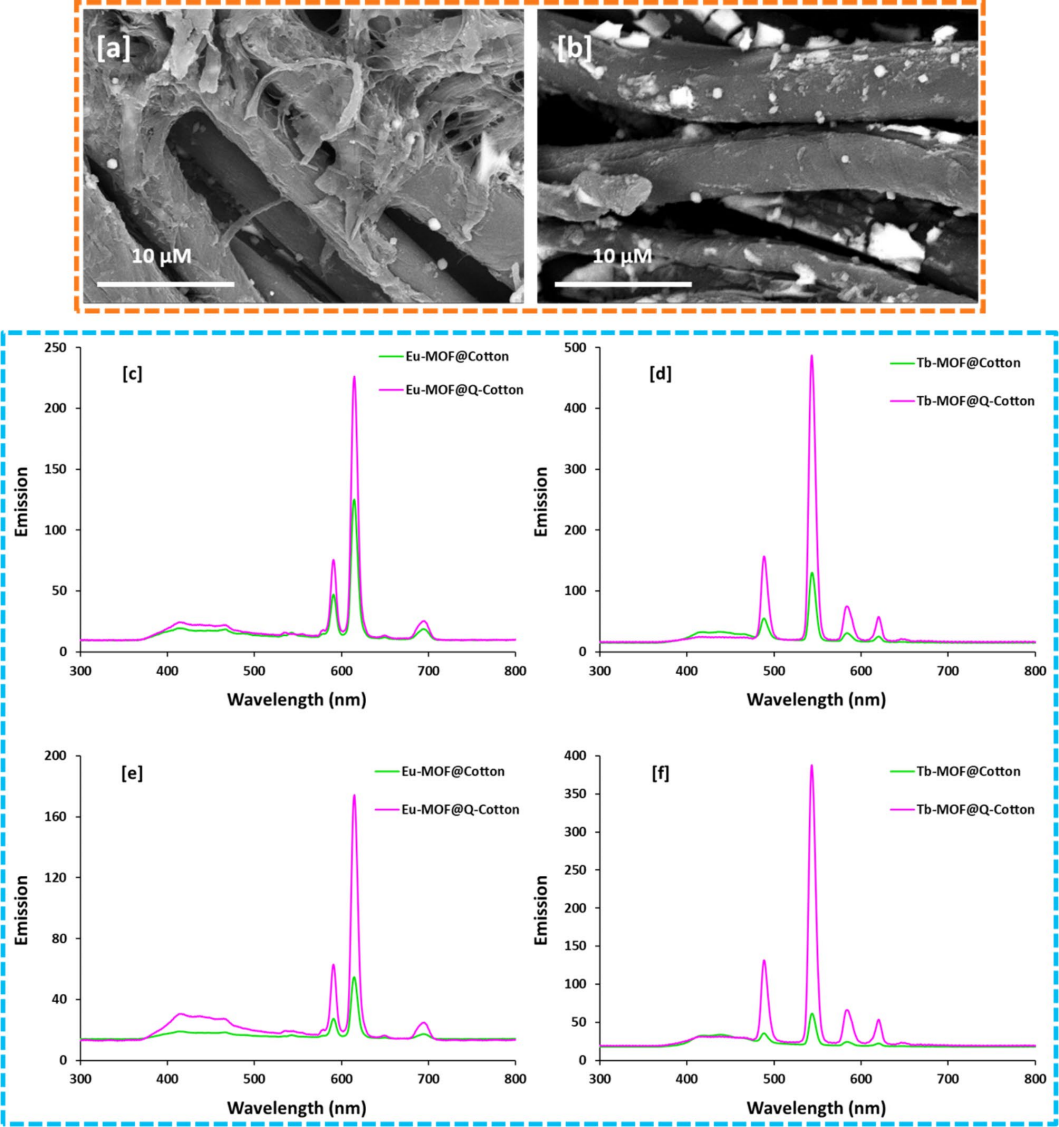
(**a**, **b**) SEM graphs for the Ln-MOF@Q-Cotton after 10 washing, (**c**, **d**, **e**, **f**) Emission spectral results for the Ln-MOF@fabric (at λ_ex_ = 270 nm) after washing, (**a**, **c**, **e**) Eu-MOF@fabric, (**b**, **d**, **f**) Tb-MOF@fabric, (**c**, **d**) 5 washings and (**e**, **f**) 10 washings.

For the wearable functional textiles, durability of the acquired functions is an important key factor for the last long applicability. The effect of washing on the durability was studied for the cationized fabrics, while the washing process was carried out 5 and 10 times according to the standard laundry process for textiles^31,45,51^. In order to check the stability of Ln-MOF within fabrics, the surface investigation of Ln-MOF@Q-cotton fabrics was examined after 10 repetitive washings. As clarified from Fig. 7a and b, the micro-deposited Ln-MOF was still observed over the Q-cotton fabrics after 10 repetitive washings, which reflected the long stand fastness of Ln-MOF after wash. Furtherly, the fastness properties for the acquired functions (UV-protection, photoluminescent and antimicrobial protection) were tested against the repetitive washing.

The photoluminescent properties for the washed Ln-MOF@fabrics were Figured in Fig. 7(c-f). The washed fabrics exhibited the three (at 592 nm, 614 nm and 698 nm) and four (490, 545, 586 and 622 nm) characterized emission bands in case of Eu-MOF and Tb-MOF, respectively. The intensities of the emission bands for Ln-MOF@fabrics were gradually decreased by washing process, while the decrement in case of uncationized cotton was significant. After 10 washing cycles, the Ln-MOF@cotton fabrics didn’t exhibit significant emission bands, while the emission bands were still clearly appeared for Ln-MOF@Q-cotton. Subsequently, the photoluminescent properties for cationized fabrics were durable even after 10 repetitive washing cycles.

The UVR protection for Ln-MOF@Q-cotton was reduced from very good level before washing to good after 10 washings as recorded in Table 4, which in turn affirmed the good UV protection standing against washing. The evaluated reduction in microbial pathogens was gradually diminished by washing, while the reduction percentage was ranged in 44–52% for Ln-MOF@Q-cotton against the different tested microbes, after 10 washing cycles. The data summarized that, some of the immobilized Ln-MOF were leached out from the fabrics during the washing process and subsequently the efficiency of the acquired functions was rationally decreased. However, the washed samples exhibited sufficient properties and hence durable UV protective, photoluminescent and antimicrobial cotton fabrics were successfully obtained by incorporation of Ln-MOF.

**Table 4:** Effect of repetitive washing on UV protection properties (AATCC) and antimicrobial activity (reduction, %) for Ln-MOF@Cotton fabrics.

	Sample	UV-protection			
		UPF	UV-A (T%)	UV-B (T%)	UPF rating
**5** **Washs**	**Eu-MOF@Q-Cotton**	31.2	2.7	9.3	*Very good*
	**Tb-MOF@Q-Cotton**	26.6	3.8	9.4	*Good*
**10 Washs**	**Eu-MOF@Q-Cotton**	24.4	3.4	10.6	*Good*
	**Tb-MOF@Q-Cotton**	21.1	3.9	10.8	*Good*

		Antimicrobial activity			
		G-ve Bacteria	G + ve Bacteria	Fungi	
		E. Coli	St. Aureus	C. Albican	
**5** **Washs**	**Eu-MOF@Q-Cotton**	66	67	68	
	**Tb-MOF@Q-Cotton**	72	69	70	
**10** **Washs**	**Eu-MOF@Q-Cotton**	51	49	52	
	**Tb-MOF@Q-Cotton**	44	49	46	

## Conclusion

The current work focused on designing of fluorescent protective cotton fabrics through immobilization of Ln(Eu & Tb)-MOF. Cationization of cotton fabrics was firstly performed, followed by the direct incorporation of Ln-MOF. Micro-deposits from Ln-MOF were clearly observed over the surface of fabrics. The designed Ln-MOF@fabrics exhibited good fluorescent emission, corresponding to the electron transition from 5D_0_ and 5D_4_ to 7F_0-4_ and 7F_3-6_ for Eu-MOF and Tb-MOF, respectively. Good and very good UV protection were recorded for Ln-MOF@cotton and Ln-MOF@Q-cotton, respectively. After Ln-MOF incorporation, all the tested microbial pathogens were reduced by 68–91% over the fabrics. All the acquired functions (fluorescence, UV-protection, antimicrobial) were improved for cotton fabrics by cationization step. The cationized fabrics showed good durability and the added functions were retained even after 10 repetitive washing cycles. The results concluded that the immobilization of Ln-MOF with cationized cotton succeeded in preparation of durable/fluorescent protective (UV protection and antimicrobial) textiles which are suitable for potential application in the military textiles.

## Data Availability

The datasets used and/or analysed during the current study available from the corresponding author on reasonable request.
